# Synergistic Antibacterial and Antibiotic Effects of Bisbenzylisoquinoline Alkaloids on Clinical Isolates of Methicillin-Resistant *Staphylococcus Aureus* (MRSA)

**DOI:** 10.3390/molecules16129819

**Published:** 2011-11-25

**Authors:** Guo-Ying Zuo, Yang Li, Tao Wang, Jun Han, Gen-Chun Wang, Yun-Ling Zhang, Wei-Dong Pan

**Affiliations:** 1 Research Center for Natural Medicines, Kunming General Hospital, PLA, Kunming 650032, China; Email: wt198331@yahoo.com.cn (T.W.); zhangyunling@126.com (Y.-L.Z.); 2 Kunming Medical College, Kunming 650032, China; Email: liyang1227@126.com (Y.L.); 3 School of Basic Medical Sciences, Yunnan Traditional Chinese Medical College, Kunming 650500, China; Email: hanzjn@126.com (J.H.); 4 The Key Laboratory of Chemistry for Natural Products of Guizhou Province and Chinese Academy of Sciences, Guiyang 550002, China

**Keywords:** anti-MRSA, synergy, bisbenzylisoquinoline alkaloid, cefazolin, FICI

## Abstract

The antibacterial activity of two bisbenzylisoquinoline alkaloids, tetrandrine (Tet) and demethyltetrandrine (d-Tet), alone and in combination with the antibiotics ampicillin (AMP), azithromycin (AZM), cefazolin (CFZ) and levofloxacin (LEV) against 10 clinical isolates of staphylococcal chromosomal cassette mec (SCCmec) III type methicillin-resistant *Staphylococcus aureus* (MRSA) was studied. Susceptibility to each agent alone was tested using a broth microdilution method. The chequerboard and time-kill tests were used for the combined evaluations. The minimal inhibitory concentrations/minimal bactericidal concentrations (MICs/MBCs, μg/mL) ranges alone were 64–128/256–1,024 for both Tet and d-Tet. Significant synergies against 90% of the isolates were observed for the Tet/CFZ combination, with their MICs being reduced by 75–94% [fractional inhibitory concentration indices (FICIs) ranged from 0.188 to 0.625], respectively. An additive bactericidal result was also observed for the Tet (d-Tet)/CFZ combination in the time-kill experiments. These results demonstrated that Tet and d-Tet enhanced the *in vitro* inhibitory efficacy of CFZ. Their potential for combinatory therapy of patients infected with MRSA warrants further pharmacological investigation.

## 1. Introduction

Clinical isolates of methicillin-resistant *Staphylococcus aureus* (MRSA) have become the most common cause of infections among the global pathogenic bacteria, and many life-threatening diseases such as endocarditis, pneumonia and toxin shock syndrome are ascribed to them. In our hospital, MRSA could be detected in over 80 percent of pneumonia sputum samples from severe and elderly patients in the intensive care unit (ICU). In the meantime, the number of new antibacterial agents approved, for example in the United States, has declined since 1983 [1]. Plants have evolved and accumulated an elaborately useful source of anti-infective drugs. The therapeutic potential of phytochemicals for the development of anti-MRSA agents has been increasingly recognized [2,3,4]. However, the reported MIC for these compounds is often in the range of 100 to 1,000 μg/mL, orders of magnitude higher than those of common broad-spectrum antibiotics obtained from bacteria or fungi [5]. Synergistic effects of natural products combined with antibiotics against infectious diseases might be a way of overcoming this deficiency [6]. Therefore, the search for novel anti-MRSA agents with novel mode of action is both urgently needed and practically achievable.

**Figure 1:**
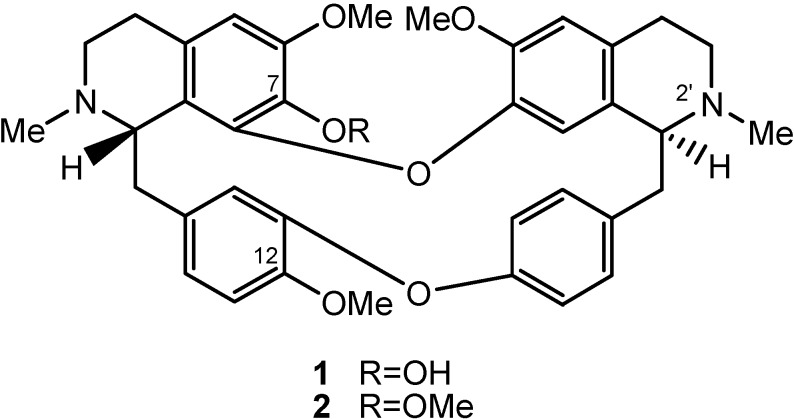
The structures of tetrandrine (Tet, R = OCH_3_, **1**) and demethyltetrandrine (d-Tet, R = OH, **2**).

In recent years, we have been engaged in a search for plant-derived compounds active against multidrug resistant (MDR) bacteria from the Traditional Chinese Medicine (TCM) repertoire [7,8,9]. We are also paying attention to the phytochemicals’ synergistic effects with routine antibiotics. We report herein for the first time the *in vitro* anti-MRSA and antibiotic synergistic effects of tetrandrine (Tet) and demethyltetrandrine (d-Tet) (Figure 1), two bisbenzylisoquinoline (BBIQ) alkaloids isolated from the Chinese drug *Stephania tetrandra* S. Moore (Fen fang chi in Chinese), on MDR clinical isolates of MRSA and their synergistic effects with four antibiotics: ampicillin (AMP), azithromycin (AZM), cefazolin (CFZ) and levofloxacin (LEV).

## 2. Results and Discussion

Anti-MRSA activities of Tet and d-Tet and the four antibiotics ampicillin (AMP), azithromycin (AZM), cefazolin (CFZ) and levofloxacin (LEV) alone against 10 clinical MRSA isolates of SCCmec III type are shown in Table 1.

**Table 1:** MICs/MBCs (μg/mL) of Tet, d-Tet and four antibiotics alone against ATCC 25923 and 10 clinical MRSA strains of SCCmec III type.

Agents ^a^	MIC/MBCs of ATCC 25923 (μg/mL)		MIC/MBCs of MRSA strains (μg/mL)		
			Range	50% ^b^	90%
Tet	64/128		64–128/256–1024	128/512	128/1024
d-Tet	32/64		64–128/256–1024	64/512	128/1024
AMP	32/64		32–128/256–512	64/512	128/512
AZM	>1,000/ nt		2,000–4,000/ nt ^c^	4,000/ nt	4,000/ nt
CFZ	64/128		128–256/ nt	128/ nt	256/ nt
LEV	4/8		8–32/32–64	16/64	16/64
VAN	0.96/1.92		0.96/1.92	0.96/1.92	0.96/1.92

^a^ Tet: tetrandine; d-Tet: demethyltetrandrine; AMP: ampicillin; CFZ: cefazolin; LEV: levofloxacin; AZM: azithromycin; VAN: vancomycin; ^b^ 50% means MIC_50_, concentration of inhibition against 50% of MRSA strains; 90% means MIC_90_, concentration of inhibition against 90% of MRSA strains; ^c^ nt: Not determined.

**Figure 2:**
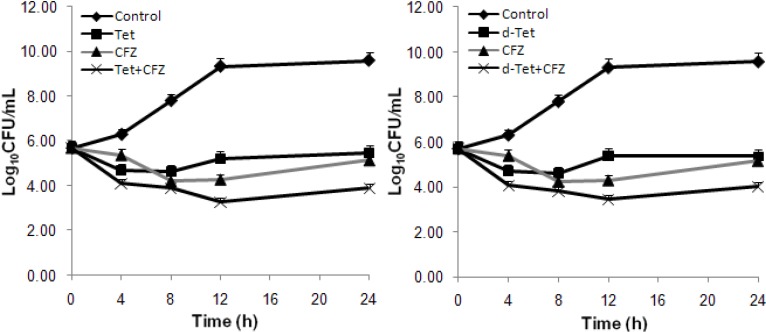
Time-kill curves of the the combination at 1× MIC concentration of tetrandrine (Tet, upper) and demethyltetrandrine (d-Tet, lower) with CFZ, respectively against MRA 276, a clinical MRSA strains of SCCmec III type. The viable cells counts reduced by 1.25 (CFZ) and 1.58 (Tet) log_10_CFU/mL (both additivity) 1.15 (CFZ) and 1.36 (d-Tet) log_10_CFU/mL (both additivity), respectively.

Synergistic effects of Tet and d-Tet with the four antibiotics against the ten MRSA isolates by the chequerboard method and the FICIs are demonstrated in Table 2. Time-killing curves of the combined effects of Tet and d-Tet with the antibiotic CFZ against MRA 276 (one of the 10 MRSA isolates) are shown in Figure 2.

**Table 2:** MICs (μg/mL) and FIC indices (FICIs) of Tet and d-Tet in combination with four antibiotics against 10 clinical MRSA strains of SCCmec III type.

Agent ^a^		Range	50%^b^	90%
Tet/AMP	MIC (μg/mL)	64–128/32–128	64/64	128/64
	Rd% ^c^	0–50/0–50	0/0	0/0
	FICI (E ^d^)	1.5(I)–2(I)	2(I)	2(I)
d-Tet/AMP	MIC (μg/mL)	64–128/32–128	64/64	128/128
	Rd%	0–50/0–0	0/0	0/0
	FICI (E)	1.5(I)–2(I)	2(I)	2(I)
Tet/AZM	MIC (μg/mL)	64–128/2000–4000	64/4000	128/4000
	Rd%	0–50/0–0	0/0	0/0
	FICI (E)	2(I)–2(I)	2(I)	2(I)
d-Tet/AZM	MIC (μg/mL)	64–128/2000–4000	64/4000	128/4000
	Rd%	0–0/0–0	0/0	0/0
	FICI (E)	2(I)–2(I)	2(I)	2(I)
Tet/CFZ	MIC (μg/mL)	8–32/16–64	8/32	32/64
	Rd%	75–94/75–88	88/75	75/75
	FICI (E)	0.25(S)–0.5(S)	0.375(S)	0.5(S)
d-Tet/CFZ	MIC (μg/mL)	8–64/16–64	16/32	32/64
	Rd%	50–88/75–94	75/75	75/75
	FICI (E)	0.188(S)–0.625(A)	0.5(A)	0.5(A)
Tet/LEV	MIC (μg/mL)	64–128/8–32	64/16	128/16
	Rd%	0–50/0–50	0/0	0/0
	FICI (E)	1.5(I)–2(I)	1.5(I)	2(I)
d-Tet/LEV	MIC (μg/mL)	32–128/8–32	64/16	128/16
	Rd%	0–50/0–50	0/0	0/0
	FICI (E)	1.5(I)–2(I)	1.5(I)	2(I)

^a^ Tet: tetrandine; d-Tet: demethyltetrandrine; AMP: ampicillin; CFZ: cefazolin; LEV: levofloxacin; AZM: azithromycin; ^b^ 50% means MIC_50_, concentration of inhibition against 50% of MRSA strains; 90% means MIC_90_, concentration of inhibition against 90% of MRSA strains; ^c^ Rd%: % of MIC reduced, Rd% = (MIC_alone_ − MIC_combined_) × 100 / MIC_alone_. Values are expressed as those of alkaloids/antibiotics; ^d^ E: Effect; A: additivity (0.5 < FICI ≤ 1); I: Indifference (1 < FICI ≤ 2); S: Synergy (FICI ≤ 0.5).

The SCCmec III type MRSA isolates are the major nosocomial strains in Asian countries and characterized by their multidrug resistance to not only β-lactams, but also to other currently used types of antibiotics [10]. MICs/MBCs (μg/mL) ranges were 64–128/256–1,024 for both Tet and d-Tet alone against all isolates. The (MICs)_50_ of Tet and d-Tet were 128 and 64 μg/mL, respectively. The agents’ order of potency followed LEV > d-Tet = AMP > Tet = CFZ >> AZM.

The chequerboard evaluation was performed with four antibiotics representing four types of antibacterial agents, including AMP (β-lactam, penicillins), AZM (macrolides), CFZ (β-lactam, cephalosporins) and LEV (fluoroquinolones). The MICs of Tet/CFZ combination were reduced by 75–94%/75–88% compared with the agents used alone, which demonstrated significant antibacterial synergy activities against 90% of the tested pathogenic strains, with the FICIs for Tet/CFZ combination ranged 0.188–0.625). Meanwhile, The MICs of d-Tet/CFZ combination were reduced by 50–88%/75–94% compared with the agents used alone, which indicated only additive activities against 50% and 90% of the same strains (Table 1 and Table 2). The rest combinations all showed indifference (FICIs 1.5–2.0). The synergy effects caused by Tet and d-Tet were obviously not the same (Table 2).

In the time-kill analyses, the combined bactericidal effects of the combinations between BBIQs and CFZ were also observed following the criterion of synergy test [11], the overall killing effects of the combinations were the best (Figure 2). The time-kill curves showed the Tet/CFZ and d-Tet/CFZ combinations resulted in killing increases of 1.25 and 1.15 log_10_CFU/mL (both additivity) of the colony counts at 24 h in comparison with that of CFZ alone, together with those of 1.58 and 1.36 log_10_CFU/mL (both additivity) of Tet and d-Tet alone, respectively (Figure 2). Hence, bactericidal efficiency of the BBIQs/CFZ combinations was much more potent than that of the antibiotics alone, which was in agreement with the bacteriostatic results in the chequerboard evaluations (Table 1 and Table 2).

We noted that the effects of BBIQs/AMP combinations (indifference) were different from those of BBIQs/CFZ combinations (synergy) though the two antibiotics are all β-lactams. Therefore, the synergistic effect of BBIQs/CFZ combinations might be dealt with the BBIQs’ inhibition against multidrug efflux pump of the MRSA strains [4,5,6]. The real mechanisms of MDR modiﬁcation remained to be investigated.

## 3. Experimental

### 3.1. Bacterial Strains and Culture Media

MRSA strains (ten isolates with SCCmec III genotype: MRA 4, 55, 123, 144, 189, 240, 276, 294, 328 and 330) were obtained and characterized from the infectious sputum samples of critically ill patients in Kunming General Hospital [7,8]. The presence of mecA gene and SCCmec genotypes were determined by multiplex PCR methods at the Kunming Institute of Virology, PLA, China, as previously reported [12]. ATCC 25923 was used as the control strain. Standard Mueller-Hinton agar and broth (MHA and MHB, Tianhe Microbial Agents Co., Hangzhou, China) were used as the bacterial culture media.

### 3.2. Antibacterial Agents

Four antibiotics representing different conventional types were purchased from the manufacturers, *i.e.*, AMP (North China Pharmaceutical Co., Ltd, Shijiazhuang, China), CFZ (Harbin Pharmaceutical Co., Ltd, Harbin, China), AZM and LEV (Yangzhijiang Pharmaceutical Co., Ltd, Taizhou, China). Vancomycin (VAN, Eli Lilly Japan K. K., Seishin Laboratories) was used as the positive control agent. Cefoxitin disks were purchased from Tiantan Biological Products Co., Ltd (Beijing, China). Tet and d-Tet were isolated and identified from the roots of *S. tetrandra* as previously reported [13]. Briefly, the ethanol (95%) root extract (200.0 g) was firstly suspended in deionized water (2,000 mL) and the pH adjusted to 3.0 with HCl (35%). The mixture was filtered and the acidic filtrate extracted with chloroform to remove lipidic non-alkaloid components. Then the acidic filtrate was made alkaline to pH 10.0 with NH_3_·H_2_O (25%) and further extracted with chloroform to afford the crude alkaloids (70.0 g), which were subjected to column chromatography on silica gel eluting with a CHCl_3_-CH_3_OH (1:0–0:1) gradient to afford four fractions (Frs. 1–4). Repeated chromatography of Fr. 2 (40.0 g) with silica gel [petroleum ether-acetone-Et_2_NH (2:1:0.2)] and Sephadex LH-20 (acetone) columns furnished Tet (4.1 g) and d-Tet (3.0 g), respectively. The purity of Tet and d-Tet were confirmed by thin-layer chromatography (TLC) which showed one spot and their contents were 97% by HPLC determination.

### 3.3. Susceptibility Testing

The minimal inhibitory concentrations/minimal bactericidal concentrations (MICs/MBCs) were determined by standardized broth microdilution techniques with starting inoculums of 5 × 10^5^ colony forming unit (CFU)/mL according to Clinical Laboratory Standards Institute (CLSI) guidelines and incubated at 35 °C for 24 h [14,15]. They were determined in duplicate, with concentrations ranging up to 4,000 μg/mL for AZM.

### 3.4. Synergy Testing

Potential anti-MRSA synergy was measured by fractional inhibitory concentration (FIC) indices (FICI) by the chequerboard method and by time-killing curves as previously reported [16]. The FIC of the combination was calculated through dividing the MIC of the BBIQs/antibiotics combination by the MIC of BBIQs or of the antibiotics alone, and the FICI was obtained by adding the FIC of BBIQs and that of antibiotics. The FICI results were interpreted as follows: FICI ≤ 0.5, synergy; 0.5 < FICI ≤ 1, additivity; and 1 < FICI ≤ 2, indifference (or no effect) and FICI >2, antagonism [16]. In the killing curves, synergy was defined as ≥2 log_10_CFU/mL increase in killing at 24 h with the combination, in comparison with the killing by the most active single drug. Additivity was defined as a 1–2 log_10_CFU/mL increase in kill with the combination in comparison with the most active single agent. Indifference was defined as ±1 log_10_CFU/mL killing or growth. Combinations that resulted in >1 log_10_CFU/mL bacterial growth in comparison with the least active single agent were considered to represent antagonism [11,17]. All experiments were performed in triplicate.

## 4. Conclusions

As the clinical MRSA strains have become an increasingly pressing global problem. Anti-MRSA synergistic effects between plant natural compounds and conventional antibacterial agents has further been demonstrated here as an alternative way of overcoming resistance to current antibiotics [18]. The results in this study showed that BBIQs enhanced the *in vitro* inhibitory efficacy of CFZ. The potential for combinatory therapy of patients infected with MRSA warrants further pharmacological investigation.
